# Clinical Significance of Potential Unidentified HLA-G Isoforms Without α1 Domain but Containing Intron 4 in Colorectal Cancer Patients

**DOI:** 10.3389/fonc.2018.00361

**Published:** 2018-09-04

**Authors:** Aifen Lin, Xia Zhang, Rui-Li Zhang, Jian-Gang Zhang, Wen-Jun Zhou, Wei-Hua Yan

**Affiliations:** ^1^Biological Resource Center, Taizhou Hospital of Zhejiang Province, Wenzhou Medical University, Linhai, China; ^2^Department of Gastrointestinal Surgery, Taizhou Hospital of Zhejiang Province, Wenzhou Medical University, Linhai, China; ^3^Medical Research Center, Taizhou Hospital of Zhejiang Province, Wenzhou Medical University, Linhai, China

**Keywords:** HLA-G, isoform, antibodies, colorectal cancer, prognosis

## Abstract

The ectopic HLA-G expression in malignancies has been extensively explored and clinical significance of the molecule was widely acknowledged. Besides previously well-documented seven isoforms (HLA-G1~-G7), other novel isoforms of HLA-G have been reported but their clinical relavenace remians evaluated. In this study, lesion HLA-G expression in 379 case-matched serial section primary colorectal cancers (CRC) were evaluated with mAb 4H84 (recognizing an epitope in HLA-G α1 domain), and mAb 5A6G7 (recognizing an epitope encoded by intron 4), respectively. Data showed that HLA-G positive staining with mAbs 4H84 and 5A6G7 was 70.7 and 60.4%, respectively. When percentage of HLA-G expression detected with mAb 4H84 subtracted that with mAb 5A6G7, the difference (_Δ_HLA-G) with negative (_Δ_HLA-G_neg_), comparable (_Δ_HLA-G_com_) and positive (_Δ_HLA-G_pos_) were observed in 64 (16.9%), 159 (42.0%), and 156 (41.2%) cases, respectively. Noteworthy, unexpected immunostaining was observed in 44 (11.6%) lesions that no staining was detected with mAb 4H84 but positive with mAb 5A6G7 (4H84^neg^5A6G7^pos^). This staining pattern was unpredictable because all seven known HLA-G isoforms containing the α 1 domain could be recognized by the mAb 4H84. Moreover, patients with _Δ_HLA-G_neg_ had obviously better survival than those with _Δ_HLA-G_com_ and _Δ_HLA-G_pos_ (*p* = 0.017), and _Δ_HLA-G could be an independent prognostic factor for CRC patients (*p* = 0.008). Our findings provides the first report that potential unidentified HLA-G isoforms is of distinct clinical significance in CRC patients.

## Introduction

The cancer promoting function of HLA-G, such as rendering comprehensive suppressive roles to various types of immune component cells and enhancing the proliferation and accumulation of immune regulatory cells have been extensively investigated (1). Different degree of aberrant ectopic expression of HLA-G in cancers has been frequently found in most of the cancers studied so far, and the conception that HLA-G expression plays critical basically and clinical parts in cancer biology or therapy was established (2).

Besides application of the clinical tumor lesions, the direct roles of HLA-G participating in cancer progression have been demonstrated in previous studies with murine models. Data obtained from xenotumor or syngeneic immunocompetent murine tumor model revealed that both HLA-G1 or HLA-G5 expression promoted tumor development by impairment of innate and adaptive immune responses but favoring the immune suppressive cells such as CD11b+Gr1+ myeloid-derived suppressor cells (MDSC) expansion (3, 4). Fortunately, these could be reversed with HLA-G and/or receptor blockade by certain antibodies. With an immunodeficient murine ovarian cancer xenotumor model, we found that HLA-G1 expressing ovarian cancer cells had stronger invasion and metastasis potential compared that with the HLA-G-negative parental cells (5). We also presented the underlying mechanisms of HLA-G in tumor progression not only related to the inhibition of NK lysis, but to the specific induction of matrix metalloproteinase-15 (MMP-15) expression (6). This finding was solidified by later studies that HLA-G5 could induce the expression and activity of MMP-2 and MMP-9 in trophoblastic cells, while knock-down the HLA-G experssion resulted in dramatically decreased expression of MMPs was also observed (7, 8).

It's well documented thus far, with the alternative splicing of the primary transcript of *HLA-G*, seven isoforms including four-membrane bound (HLA-G1, -G2,-G3, and -G4) and three soluble (HLA-G5, -G6, and -G7) molecules were validated. Each unique HLA-G isoform contains one to three extracellular globular domains encoded by exon 2 (α 1), exon 3 (α2) and exon 4 (α3) respectively, and presence or absence of residues encoded by intronic sequences (IMGT/HLA database). However, these seven isoforms all contain the extracellular α 1 domain (9, 10). Among these isoforms, HLA-G1 and soluble HLA-G5 molecule have been studied more extensively due to the commercially available antibodies. However, it's reasonable that other novel unrecognized HLA-G isoforms remains unveiled. Indeed, based on immunohistochemical and deep sequencing, an important study by Tronik-Le Roux et al. (11) recently revealed that unrecognized novel HLA-G isoforms such as isoforms without α1 domain were presented in renal cancer samples. The findings raises the questions whether these novel HLA-G proteins are of clinical significance.

In our study, mAbs 4H84 and 5A6G7 were used to analyze the HLA-G expression by immunohistochemistry in primary colorectal cancer (CRC) samples. The percentage of HLA-G expression in tumor cells by mAbs 4H84 and 5A6G7 were compared, and the difference of the HLA-G expression (_Δ_HLA-G) was obtained by the percentage of HLA-G expression detected with HLA-G mAb 4H84 subtracted that with mAb 5A6G7. _Δ_HLA-G with negative (_Δ_HLA-G_neg_), comparable (_Δ_HLA-G_com_) and positive (_Δ_HLA-G_pos_) were observed. Our findings revealed that patients with _Δ_HLA-G_neg_ had much better survival than patients with _Δ_HLA-G_com_ and _Δ_HLA-G_pos_, and the _Δ_HLA-G could be an independent prognostic factor for CRC patients.

## Materials and methods

### CRC patients

A cohort of 379 consecutive primary CRC lesions were provided by Tissue Bank of Taizhou Hospital of Zhejiang Province (National human genetic resources platform of China YCZYPT[2017]02). Patients were diagnosed and treated at the department of Gastrointestinal Surgery in Taizhou Hospital of Zhejiang Province from November 2004 to September 2012.

The clinical stage including stage I (*n* = 86), II (*n* = 114), III (*n* = 169), and IV (*n* = 11) respectively, were classified according to the UICC and the AJCC 7th TNM staging system (12). Details of the clinical history of the patients was recorded and the last follow-up was performed at April,15th, 2014. During the period, 38 patients was lost follow-up in the cohort. Overall survival was calculated from the date of surgical operation to the event (patient death, *n* = 113) or censored (last follow-up, *n* = 228) with the median follow-up of 45.0 months. 113 cancer-related deaths was observed in 10 stage I, 26 stage II, 71 stage III and 6 stage IV CRC patients. Informed written consent was provided by all participated patients and the study protocol was approved by the institutional ethic review board, Taizhou Hospital of Zhejiang Province.

### Immunohistochemistry and staining evaluation

Immunohistochemistry analysis for HLA-G expression was performed for each case-matched serial section primary CRC lesions on 4-μm paraffin-embedded sections according to our previous study with the two anti-HLA-G murine monoclonal antibodies (mAb) 4H84 (1:500, Exbio, Prague, Czech Republic) and 5A6G7 (1:500, Exbio, Prague, Czech Republic), respectively (13, 14). mAb 4H84 (IgG1) was immunized with amino acids 61–83 in the HLA-G alpha 1 domain, which recognizes an epitope located in the α 1 domain in denatured heavy chain to all α 1 domain containing HLA-G isoforms such as HLA-G1~HLA-G7. mAb 5A6G7 (IgG1) was immunized with C-terminal amino acid sequence (22-mer) of HLA-G5 and -G6 proteins coupled to ovalbumin, which recognizes HLA-G isoforms such as HLA-G5 and -G6 encoded by the retained intron 4 (IMGT/HLA database), but not cross react with the HLA-G1 isoform.

The percentage of HLA-G expression was evaluated by two reviewers who were blind to the patient clinicopathological information. The percentage of HLA-G positive tumor cells was based on the presence of HLA-G staining while irrespective the staining intensity. HLA-G positive CRC cells >5% in a section was considered as positive (15).

Difference of the percentage of HLA-G positve tumor cells (_Δ_HLA-G) in the case-matched CRC samples was calculated by the percentage of HLA-G expression detected with mAb 4H84 subtracted that with mAb 5A6G7. According to the value of _Δ_HLA-G, three groups were obtained as follows: _Δ_HLA-G_neg_ (_Δ_HLA-G> −5.0%), _Δ_HLA-G_com_ (−5.0%≤_Δ_HLA-G≤5.0%), and _Δ_HLA-G_pos_ (_Δ_HLA-G>5.0%).

### Statistical analysis

Pearson chi-square test was used for categorical data analysis. Survival probability analysis were performed with Kaplan-Meier method and log-rank test. Cox proportional hazards model was used for multivariate analysis. *P* < 0.05 (two-tailed) was considered as statistically significant. All statistical analysis was performed with the SPSS 13.0 software (SPSS, Inc., Chicago, IL, USA).

## Results

### Expression of HLA-G probed with mAbs 4H84 and 5A6G7 in CRC

**Figure 1:**
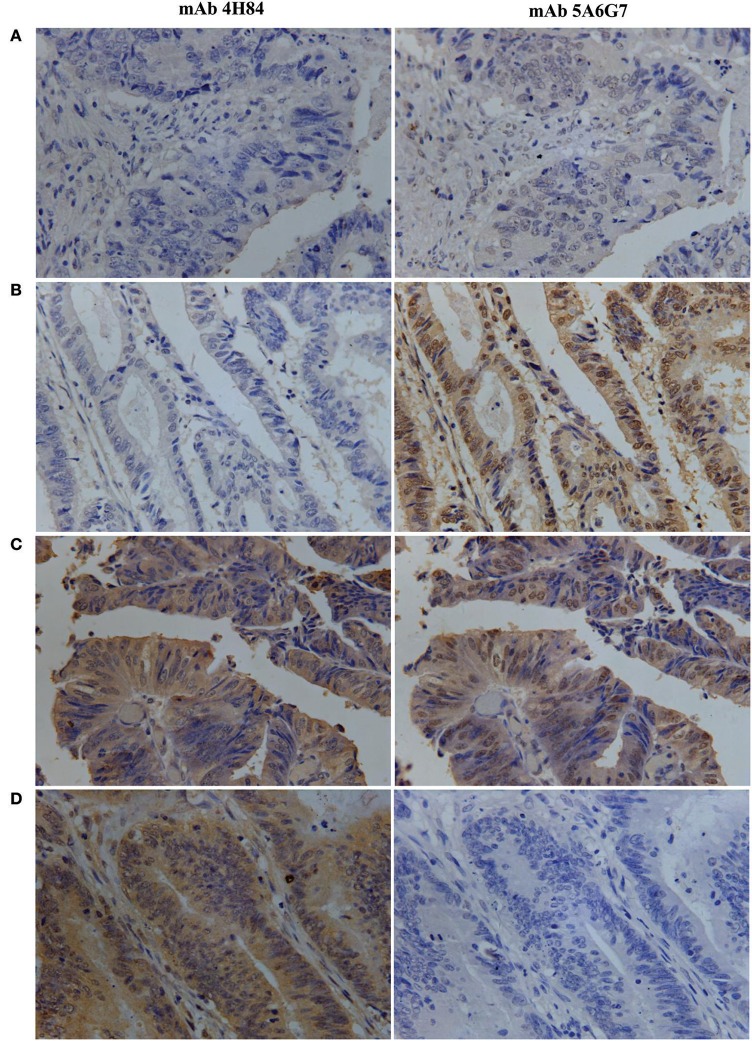
Representative immunohistochemistry analysis of HLA-G expression patterns in primary CRC serial section lesions with mAbs 4H84 and 5A6G7. **(A)** mAbs 4H84_neg_5A6G7_neg_; **(B)** mAbs 4H84_neg_ 5A6G7_pos_; **(C)** mAbs 4H84_pos_5A6G7_pos_; and **(D)** mAbs 4H84_pos_5A6G7_neg_. (400×).

The IHC immunostaining pattern with mAbs 4H84 and 5A6G7 was shown in Figure 1. In these case-matched serial section primary CRC lesions, positive HLA-G expression was observed in 70.7% (268/379) with mAb 4H84 and in 60.4% (219/379) with mAb 5A6G7 in CRC patients. The percentage of the HLA-G expression detected with both antibodies ranges from negative to 99%. Neither the HLA-G expression status detected with mAb 4H84, nor with mAb 5A6G7 was statistical significantly related to the clinicopathological parameters such as gender, age, TNM status and AJCC clinical disease stage of CRC patients (Table 1).

**Table 1:** HLA-G expression detected with mAbs 4H84 and 5A6G7 related to the clinical parameters in CRC patients.

**Variables**	**Cases**	**HLA-G (mAb 4H84)**			**HLA-G(mAb 5A6G7)**		
		**Neg**.	**Pos. (%)**	p^*^	**Neg**.	**Pos. (%)**	p^*^
Gender							
Male	214	69	145 (67.8%)	0.150	95	119 (55.6%)	0.329
Female	165	42	123 (74.5%)		65	100 (60.6%)	
Age							
≤ median (66 ys)	188	52	136 (72.3%)	0.490	78	110 (58.5%)	0.776
>median	191	59	132 (69.1%)		82	109 (57.1%)	
TNM stage							
Tumor status							
T_1+2_	108	26	82 (75.9%)	0.016	39	69 (63.9%)	0.110
T_3_	256	79	177 (69.1%)		117	139 (54.3%)	
T_4_	15	6	9 (60.0%)		4	11 (73.3%)	
Nodal status							
N_0_	201	57	144 (71.6%)	0.870	82	119 (59.2%)	0.421
N_1_	109	34	75 (68.8%)		44	65 (54.3%)	
N_2_	69	20	49 (71.0%)		34	35 (73.3%)	
Metastasis status							
M_0_	368	110	258 (70.1%)	0.135	156	212 (57.6%)	0.690
M_1_	11	1	10 (90.9%)		4	7 (63.6%)	
Disease stage							
I	85	23	62 (72.9%)	0.434	28	57 (67.1%)	0.207
II	114	34	80 (70.2%)		54	60 (52.6%)	
III	169	53	116 (68.6%)		74	95 (56.2%)	
IV	11	1	10 (90.9%)		4	7 (63.6%)	

* *Comparison of HLA-G expression status between or among each variable using the Pearson chi-square test. TNM, lymph-node-metastasis and stage according to the TNM classification*.

Be noted, differential HLA-G expression was commonly observed in the case-matched CRC samples between the samples detected with mAbs 4H84 and 5A6G7. In this CRC cohort, _Δ_HLA-G_neg_ in 64 (16.9%), _Δ_HLA-G_com_ in 159 (42.0%), and _Δ_HLA-G_pos_ in 156 (41.2%) CRC cases was observed. Among the _Δ_HLA-G_neg_ group, unexpected immunostaining was observed in 44 (11.6%) case-matched CRC lesions that no staining was detected with the mAbs 4H84 but with the 5A6G7 (mAbs 4H84^neg^5A6G7^pos^). The data were unpredictable beacuse all seven known α1 domain containing HLA-G isoforms could be recognized by the mAb 4H84. However, both _Δ_HLA-G and its subgroups mAbs 4H84^neg^5A6G7^pos^ and 4H84 ^pos^5A6G7 ^neg^ status was unrelated to the clinicopathological parameters of CRC patients (Table 2).

**Table 2:** Difference (_Δ_HLA-G) with mAbs 4H84 and 5A6G7 related to the clinical parameters in CRC patients.

**Variables**	**Cases**	_**Δ**_HLA-G^*^				**HLA-G (mAb 4H84 vs. 5A6G7)**			
		**Neg**.	**Com**	**Pos**	p^**^	**Cases**	**4H84^neg^ 5A6G7^pos^**	**4H84^pos^ 5A6G7^neg^**	p^**^
Total	379	64	159	156		137	44	93	
Gender									
Male	214	35	96	83	0.418	76	25	51	0.828
Female	165	29	63	73		61	19	42	
Age									
≤ median (66 ys)	188	32	79	77	0.996	68	21	47	0.759
>median	191	32	80	79		69	23	46	
TNM stage									
Tumor status									
T_1+2_	108	19	47	42	0.821	33	10	23	0.175
T_3_	256	41	107	108		100	31	69	
T_4_	15	4	5	6		4	3	1	
Nodal status									
N_0_	201	33	88	80	0.634	65	20	45	0.356
N_1_	109	22	40	47		46	18	28	
N_2_	69	9	31	29		26	6	20	
Metastasis									
M_0_	368	63	154	151	0.782	134	44	90	0.228
M_1_	11	1	5	5		3	0	3	
Disease stage									
I	85	14	41	30	0.885	23	9	14	0.417
II	114	19	47	48		42	11	31	
III	169	30	66	73		69	24	45	
IV	11	1	5	5		3	0	3	

* _Δ_HLA-G: the difference of the percentage of HLA-G expression detected with mAb 4H84 subtracted that with mAb 5A6G7. _Δ_HLA-G_neg:Δ_HLA-G >−5.0%; _Δ_HLA-G_com_:−5.0%≤_Δ_HLA-G≤5.0%; _Δ_HLA-G_pos_: _Δ_HLA-G > 5.0%.

** *Comparison of HLA-G expression status between or among each variable using the Pearson chi-square test. TNM, lymph-node-metastasis and stage according to the TNM classification*.

The mAb 5A6G7 specifically recognize an epitope located in the C-terminal amino acid sequence of HLA-G isoforms such as HLA-G5 and -G6. However, being with the α 1 domain, HLA-G5 and -G6 could also be recognized by the mAb 4H84. Therefore, to our prevailing knowledge, absence of mAb 4H84 labeling generally considered as the lack of HLA-G antigen expression. Noteworthy, the unexpected immunostaining pattern mAbs 4H84^neg^5A6G7^pos^ now sounds reasonable by the recent findings by the study Tronik-Le Roux et al. (11) that novel HLA-G isoforms lacks the α1 domain indeed exist in renal cancers.

### Significance of the _Δ_HLA-G status to CRC patient survival

Herein, Log-rank Mantel-Cox analysis of _Δ_HLA-G status and clinical parameters in CRC patient survival was evaluated. Data showed that, in addition to CRC patient tumor status, nodal status, metastasis status and AJCC clinical disease stage, both _Δ_HLA-G and its subgroups mAbs 4H84^neg^5A6G7^pos^ and 4H84 ^pos^5A6G7 ^neg^ status were significantly related to survival (Table 3).

**Table 3:** Log-rank Mantel-Cox analysis of clinical parameters in survival in CRC patients.

**Variables**		**No. total**	**No. events**	**Mean survival**	**95% CI**	***p*-value**
Sex	Male	192	65	72.0	66.2–77.7	0.927
	Female	147	48	73.4	66.6–80.1	
Age	≤ 66 ys	164	55	73.4	67.1–79.6	0.755
	> 66 ys	175	58	71.6	65.4–77.8	
Tumor status	T_1+2_	97	18	84.5	77.4–91.6	0.001
	T_3_	228	87	67.6	62.1–73.1	
	T_4_	14	8	60.6	40.1–81.2	
Nodal status	N_0_	176	37	84.4	79.2–89.6	<0.001
	N_1_	101	44	62.4	54.1–70.7	
	N_2_	62	32	53.6	42.3–65.0	
Metastasis status	M_0_	328	107	73.4	68.8–77.8	0.089
	M_1_	11	6	51.9	25.2–78.7	
Clinical stage	I	76	10	86.3	79.7–92.9	<0.001
	II	98	26	80.4	73.2–87.7	
	III	154	71	58.7	51.8–65.7	
	IV	11	6	51.9	25.1–78.7	
HLA-G (mAb 4H84)	Neg	98	29	76.7	69.0–84.5	0.250
	Pos	241	84	70.7	65.4–76.0	
HLA-G (mAb 5A6G7)	Neg	142	54	69.5	62.9–76.2	0.268
	Pos	197	59	74.9	69.0–80.8	
_Δ_HLA-G status^*^						
	_Δ_HLA-G_neg_	57	10	87.9	79.4–96.4	0.017
	_Δ_HLA-G_com_	143	48	71.0	64.0–77.9	
	_Δ_HLA-G_pos_	139	55	67.2	60.3–74.1	
4H84^neg^5A6G7^pos^	_/_	40	8	85.5	74.8–96.2	0.046
4H84^pos^5A6G7^neg^	_/_	84	33	68.1	59.2–77.0	

* *_Δ_HLA-G: the difference of the percentage of HLA-G expression detected with mAb 4H84 subtracted that with mAb 5A6G7. _Δ_HLA-G_neg:Δ_HLA-G >−5.0%; _Δ_HLA-G_com_:−5.0%≤_Δ_HLA-G≤5.0%; _Δ_HLA-G_pos_: _Δ_HLA-G > 5.0%*.

**Figure 2:**
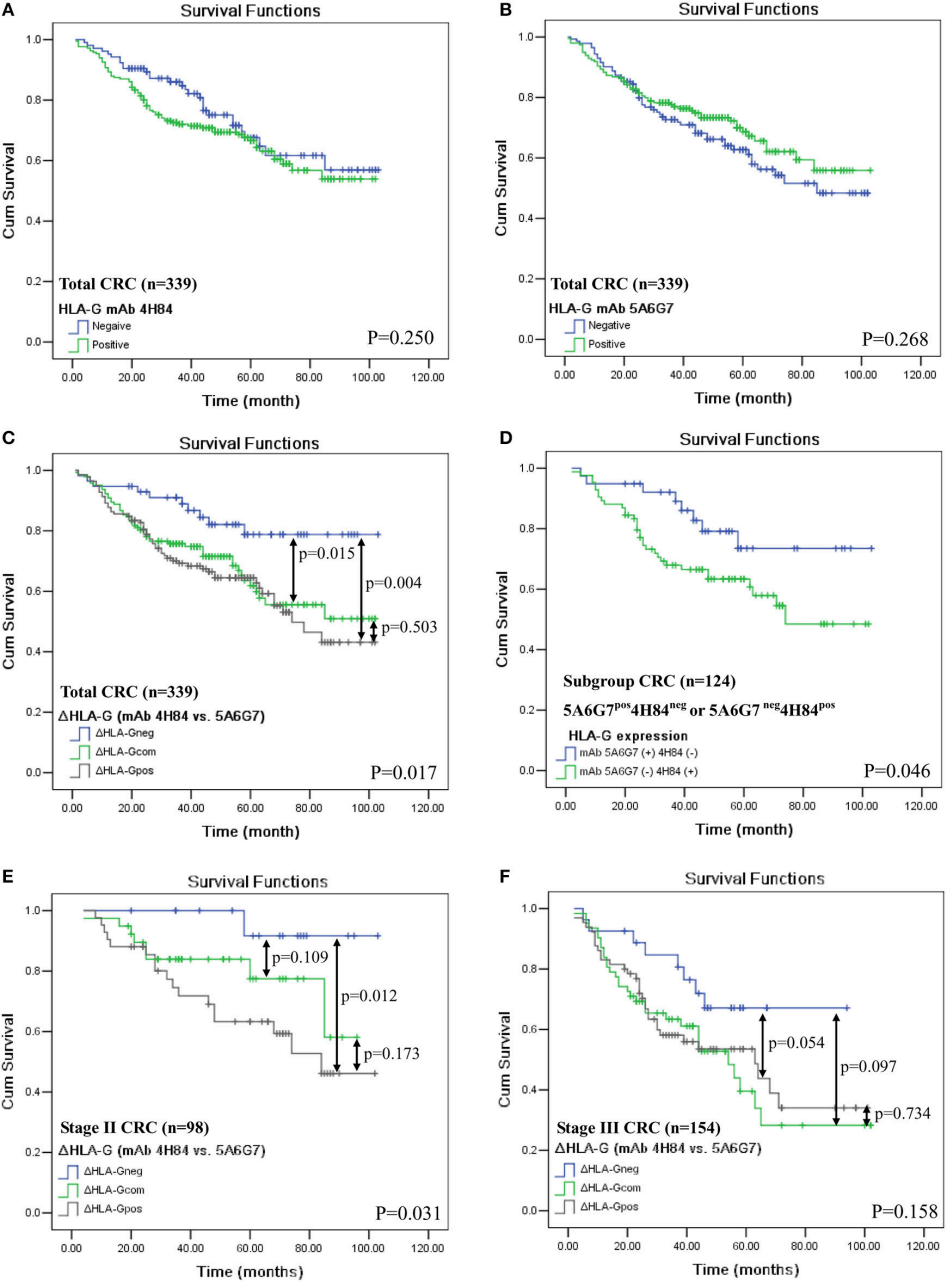
Kaplan-Meier survival analysis of HLA-G expression status with mAbs 4H84 or 5A6G7 in CRC patients. **(A)** Comparison between HLA-G_neg_ and HLA-G_pos_ with mAb 4H84 in the whole cohort of CRC patients (*p* = 0.250); **(B)** between HLA-G_neg_ and HLA-G_pos_ with mAb 5A6G7 in the whole cohort of CRC patients (*p* = 0.268); **(C)** among patient with _Δ_HLA-G_neg_, _Δ_HLA-G_com_ and _Δ_HLA-G_pos_ (*p* = 0.017) in the whole cohort of CRC patients. **(D)** between patients with HLA-G status of mAbs 5A6G7^pos^4H84^neg^ and with mAbs 5A6G7^neg^4H84^pos^ (*p* = 0.046); **(E)** among AJCC stage II CRC patient with _Δ_HLA-G_neg_, _Δ_HLA-G_com_ and _Δ_HLA-G_pos_ (*p* = 0.031); and **(F)** among AJCC stage III CRC patient with _Δ_HLA-G_neg_, _Δ_HLA-G_com_ and _Δ_HLA-G_pos_ (*p* = 0.158). Note: *p*-value in double-head arrow represents the comparison between subgroups of the CRC patients indicated.

In addition, HLA-G expression status detected by either mAbs 4H84 (*p* = 0.250, Figure 2A) or 5A6G7 (*p* = 0.268, Figure 2B) were not associated with CRC patient survival. However, in the whole CRC cohort, _Δ_HLA-G status was dramatically relevant to the patient survival (*p* = 0.017, Figure 2C). The mean survival for _Δ_HLA-G_neg_ was 87.9 months (*n* = 57; 95% CI: 79.4–96.4), _Δ_HLA-G_com_ was 71.0 months (*n* = 143; 95% CI: 64.0–77.9), and _Δ_HLA-G_pos_ was 67.2 months (*n* = 139; 95% CI: 60.3–74.1) respectively, where patients with _Δ_HLA-G_neg_ had a better outcome than that of patients with _Δ_HLA-G_com_ and _Δ_HLA-G_pos_. Interestingly, when compared the survival between the patients with mAbs 4H84^neg^5A6G7^pos^ (*n* = 40) and mAbs 4H84 ^pos^5A6G7^neg^ (*n* = 84), we found that patients with mAbs 4H84^neg^5A6G7^pos^ had a better survival than that of patients with mAbs 4H84^pos^5A6G7^neg^ (*p* = 0.046, Figure 2D). The mean survival for mAbs 4H84^neg^5A6G7^pos^ was 85.5 months (95% CI: 74.8–96.2), and mAbs 4H84^pos^5A6G7^neg^ was 68.1 months (95% CI: 59.2–77.0), respectively.

To mitigate the heterogeneity of the samples on the prognistic value of _Δ_HLA-G, we futher analyzed the relevance of _Δ_HLA-G status to survival for patients with particular AJCC stages. The survival analysis was performed for patients with AJCC stage II and III, because limited evens were occurred in patietns with AJCC stage I (N_event_ = 10) and stage IV (N_event_ = 6). Data showed that, among patients with AJCC stage II, _Δ_HLA-G status was also obivously associated with the patient survival, the mean survival for _Δ_HLA-G_neg_ (*n* = 17)_, Δ_HLA-G_com_ (*n* = 39), and _Δ_HLA-G_pos_ (*n* = 42) was 99.3, 79.1, and 70.5 months, respectively (*p* = 0.031). For the subgroups of stage II patients, significant difference for survival was also observed between the patients with _Δ_HLA-G_neg_ vs. _Δ_HLA-G_pos_ (*p* = 0.012; Figure 2E). Among patients with AJCC stage III, though does not reach the statistical significance, the mean survival for _Δ_HLA-G_neg_ (*n* = 27, 72.7 months) was much longer than patients with_, Δ_HLA-G_com_ (*n* = 62, 53.6 months) and _Δ_HLA-G_pos_ (*n* = 65, 56.7 months; *p* = 0.158, Figure 2F).

### Value of _Δ_HLA-G status on prognosis for CRC patients

Finally, the prognostic value of the _Δ_HLA-G combined with CRC patient clinical parameters was analyzed (Table 4). By univariate analysis, primary tumor burden (T_3+4_ vs. T_1+2_, *HR* = 2.504, *p* < 0.001), regional lymph node (N_1+2_ vs. N_0_, *HR* = 3.064, *p* < 0.001), distant metastases (M_1_ vs. M_0_, *HR* = 2.009, *p* = 0.097), and clinical disease stage (III + IV vs. I + II, *HR* = 3.142, *p* < 0.001) was markedly associated with worse prognosis; However, _Δ_HLA-G _neg_ (_Δ_HLA-G _neg_ vs. _Δ_HLA-G _com+pos_, *HR* = 0.414, *p* = 0.008) was obviously associated with a better prognosis. Furthermore, by multivariate analysis, data showed that primary tumor (*p* = 0.030) and _Δ_HLA-G status (*p* = 0.008) remain statistic significantly, indicating that _Δ_HLA-G status could be an independent prognostic factor for CRC patients.

**Table 4:** Cox proportional hazards model analysis of multi-variables in survival for CRC patients.

**Variables**	**Categories**	**Univariate analysis**		**Multivariate analysis**	
		**HR (95% CI)**	***P***	**HR (95% CI)**	***P***
Sex	Male vs. Female	0.983 (0.667–1.427)	0.927		
Age (years)	>66 vs. ≤ 66	1.060 (0.733–1.534)	0.756		
Tumor status	T_3+4_ vs. T_1+2_	2.504 (1.512–4.147)	<0.001	1.793 (1.059–3.035)	0.030
Nodal status	N_1+2_ vs. N_0_	3.064 (2.062–4.554)	<0.001	0.919 (0.107–7.921)	0.919
Metastasis status	M_1_ vs.M_0_	2.009 (0.881–4.580)	0.097	1.047 (0.419–2.612)	0.922
Clinical stage	III+IV vs. I+II	3.142 (2.108–4.683)	<0.001	2.913 (0.322–26.34)	0.341
HLA-G (mAb 4H84)	Pos vs. Neg	1.267 (0.830–1.933)	0.272		
HLA-G (mAb 5A6G7)	Pos vs. Neg	0.812 (0.561–1.175)	0.270		
_Δ_HLA-G status^*^	_Δ_HLA-G _neg_ vs. _Δ_HLA-G _com+pos_	0.414 (0.216–0.792)	0.008	0.416 (0.217–0.798)	0.008

HR, hazard ratio; 95% CI = 95% confidence interval.

* *_Δ_HLA-G: the difference of the percentage of HLA-G expression detected with mAb 4H84 subtracted that with mAb 5A6G7. _Δ_HLA-G_neg:Δ_HLA-G > −5.0%; _Δ_HLA-G_com_: −5.0%≤_Δ_HLA-G≤5.0%; _Δ_HLA-G_pos_: _Δ_HLA-G > 5.0%*.

## Discussion

Since HLA-G was firstly found in cytotrophoblast in 1990 (16), immune regulation functions of HLA-G have been investigated extensively, and important roles of HLA-G in the maintenance for the fetal-maternal immunotolerance were established (17). However, with the HLA-G expression was observed in cancers for the first time in 1998, numerous studies have been carried out focusing on the basic biology and clinical significance of HLA-G in malignancies (2, 18). In clinical settings and pre-clinical murine models, HLA-G expression was strongly related to the capability of tumor cell invasiveness and metastasis, to advanced disease stage and poor survival in cancer patients (1, 13). Thus far, the importance of HLA-G in cancer promotion and progression has been widely acknowledged. Consequently, HLA-G as cancer immunotherapy target was proposed.

One of the distinct features of *HLA-G* is that, by gene alternative splicing, its primary transcript could generate at least seven isoforms (HLA-G1~HLA-G7) (9). However, it's reasonable more novel unrecognized HLA-G isoforms remains unveiled because different reading frame could be formed by the gene splicing. This is indeed in case of HLA-G as reported in a recent study (11). In that study, HLA-G expression in renal cancer lesions (RCC) was evaluated by immunohistochemistry (IHC) with different HLA-G antibodies including mAbs 4H84 and 5A6G7. Unexpectedly, in some RCC cases, IHC results showed that no immunostaining was detected with the mAb 4H84 but strongly with mAb 5A6G7 (mAbs 4H84^neg^5A6G7^pos^). The staining pattern seems controversy to our prevailing knowledge that absence of mAb 4H84 labeling generally accounts for the lack of HLA-G expression. This is based on the fact that all previously identified seven HLA-G isoforms contains α 1 domain and could be detected by the mAb 4H84.

In this context, we presented similar puzzled data of staining pattern (mAbs 4H84^neg^5A6G7^pos^) on the case-matched lung and ovarian cancers and lead to hot discussion by the participants during the 7th international conference on HLA-G (Paris, 2015. http://www.hlag2015.sitew.fr/#Program.C). Noteworthy, now the unpredictable and unexpected immunostaining pattern could be explained by the findings revealed by Tronik-Le Roux et al. (11), that unrecognized HLA-G isoforms heretofore such as α1 domain-absent isoforms were presented in CRC samples. The very instructive findings raises the questions whether these novel HLA-G proteins are of clinical significance because similar, distinct or opposing functions was observed among different isoforms.

Substantial previous evidence has proved that HLA-G is a crucial tumor-driven immune escape factor involved in alterating the anti-tumor responses of immune contexture of solid tumors like CRC, and thus in determination of the fate of tumor development and patient clinical outcome (1). Through binding to the immune inhibitory receptors (particularly ILT2 and/or ILT4) expressed on various immune cells, HLA-G could directly or indirectly impair anti-tumor immune responses (2). Accumulating data revealed that HLA-G has comprehensive immune suppressive functions to T cells, B cells, NK cells and DCs, and to enhance the generation of immune regulatory cells such as MDSCs, DC-10 and M2-macrophages (19–23). Among HLA-G isofroms, the immune suppression mediated by HLA-G1 and HLA-G5 isoforms was well documented by different research groups, including ours. We found that HLA-G1 expressed in ovarian cancer cell lines NIH:OVCAR-3 and HO-8910 (6, 24), hepatocellular carcinoma cell line Hep G2 (25), lung adenocarcinoma cell line A549 (26) could dramatically decrease the NK cell cytolysis. We also found that by blocking the HLA-G with mAb 87G or its receptor ILT2 expressed on the effector NK-92 cell line could significantly reverse the NK cell lysis function (24, 26). Furthermore, HLA-G1 and HLA-G5 isoforms in NK cell cytolysis suppression is dependent on the level of both HLA-G1 and HLA-G5 expression. Compared to HLA-G1, HLA-G5 has a more potent inhibition effect on the NK cytolysis. Moreover, HLA-G1 and HLA-G5 isoforms have an additive effect on NK cytolysis suppression (27, 28). HLA-G1/ILT2 engagement on T cells could result in inhibition of CD4+ T cell alloproliferation and CD8+ and Vγ9Vδ2 T cell cytolysis and IFN-γ production (29–31). HLA-G5/ILT2 mediated signal was observed to supress B cell immunoglobulin production, and HLA-G5/ILT4 interaction impair phagocytosis and reactive oxygen species production of neutrophils (32, 33). Other functions such as impairment of the expression and function of chemokine receptors in T cells, NK cells and B cells mediated by HLA-G5/ILT2 signaling pathway was also reported (34).

As expected that, like other HLA antigens, subtle structural alterations in HLA-G molecules can have dramatic effects on innate and adaptive immune modulation, and consequently, related to different clinical significance (35, 36). In this scenario, recent studies revealed that the nature of the bound peptide could influence the substructure and stability of HLA-G, and a single amino acid exchange in the α2 domain in allelic HLA-G subtypes of HLA-G^*^01:01 and HLA-G^*^01:04 has obivously different capability in NK cell lysis inhibition (36, 37). HLA-G3 was found less efficient than HLA-G1 at inducing HLA-E surface expression (38). Moreover, unlike HLA-G1, HLA-G3 could not inhibit human monocyte/macrophage-mediated swine endothelial cell lysis or NK cells against K562 cells (39, 40). These findings indicated that distinct structure of HLA-G molecules or isoforms features with unique biological functions and could impact on its recognition by receptors of both innate and adaptive immune systems.

To evaluate whether different structural HLA-G molecaules have different clinical relavence, in this study, HLA-G expression in 379 case-matched primary CRC lesions were analyzed by IHC with mAb 4H84 and mAb 5A6G7. Different percentage of HLA-G expression was commonly observed between the case-matched serial sections when using the mAb 4H84 and mAb 5A6G7. Furthermore, clinical relevance of the difference of HLA-G expression (_Δ_HLA-G) between mAbs 4H84 and 5A6G7 was evaluated. Our data revealed that lower percentage of HLA-G expression detected with mAb 4H84 than mAb 5A6G7 (_Δ_HLA-G_neg_) was found in 16.9% (64/379), comparable percentage detected with both antibodies (_Δ_HLA-G_com_) was found in 42.0% (159/379), higher percentage of HLA-G expression detected with mAb 4H84 than mAb 5A6G7 (_Δ_HLA-G_pos_) was found in 41.2% (156/379) of CRC samples, respectively. Patients with _Δ_HLA-G_neg_ had significantly better survival than those with _Δ_HLA-G_com_ and _Δ_HLA-G_pos_ CRC patients (*p* = 0.017). A better predictive value of _Δ_HLA-G_neg_ was also observed for patients with either AJCC stage II or III. Moreover, patients with staining pattern 4H84^neg^5A6G7^pos^ had a better survival than that of patients with 4H84^pos^5A6G7^neg^ (*p* = 0.046). Multivariate analysis by Cox proportional hazards model also showed that _Δ_HLA-G could be an independent prognostic factor for CRC patients (*p* = 0.008).

Different clinical significance of HLA-G expression in cancers detected with mAb 4H84 and mAb 5A6G7 was also reported in previous studies. HLA-G expression detected with mAb 4H84 was found strongly relative to clinical stage and poor prognosis in lung cancers, while no such significance was observed for HLA-G5/-G6 detected with mAb 5A6G7 (26, 41). Unlike detected with mAb 4H84, HLA-G expression detected with mAb 5A6G7 was also found to unrelated to clinical parameters such as FIGO stage and prognosis in ovarian cancers (14, 42). Thus, the immunostaining pattern for HLA-G expression with 4H84^neg^5A6G7^pos^ strengthen the existence of novel α1 domain-absent HLA-G isoforms which could not be probed by the mAb 4H84. However, their specific function in physiological and pathological conditions still remains to be determined. To this end, being lack of currently available commercial antibodies for those novel HLA-G isoforms, a large campaign to develop new antibodies is extremely necessary and even inevitable.

Summary, our preliminary findings revealed that novel unidentified HLA-G isoforms recognized by mAbs 5A6G7 but not 4H84 might be existed in colorectal cancers. Moreover, HLA-G isoforms recognized by mAb 5A6G7 is of different clinical significant from that of the isoforms recognized by mAb 4H84. In the era of the precision medicine, the inter- and intra-tumor heterogeneity caused by complexity of the validated and even novel HLA-G isoforms, interpretation of the significance of HLA-G in cancers must be with extreme caution before their distinct roles are elucidated.

## Author contributions

AL and W-HY: study design; XZ and AL: performed experiments; R-LZ, J-GZ, and W-JZ: material support and data acquisition; AL and W-HY: performed statistical analysis and drafted the manuscript. All authors read and approved the final manuscript.

### Conflict of interest statement

The authors declare that the research was conducted in the absence of any commercial or financial relationships that could be construed as a potential conflict of interest.
